# Bioresorbable porous β-tricalcium phosphate chelate-setting cements with poly(lactic-co-glycolic acid) particles as pore-forming agent: fabrication, material properties, cytotoxicity, and *in vivo* evaluation

**DOI:** 10.1080/14686996.2021.1936628

**Published:** 2021-06-24

**Authors:** Akihiro Ando, Maho Kamikura, Yuko Takeoka, Masahiro Rikukawa, Kazuaki Nakano, Masaki Nagaya, Hiroshi Nagashima, Mamoru Aizawa

**Affiliations:** a Department of Applied Chemistry, School of Science and Technology, Meiji University, Kanagawa, Japan; b Department of Materials and Life Sciences, Faculty of Science and Technology, Sophia University, Tokyo, Japan; c Meiji University International Institute for Bio-Resource Research, Meiji University, Kanagawa, Japan; d Department of Life Sciences, School of Agriculture, Meiji University, Kanagawa, Japan; e Meiji University International Institute for Materials with Life Functions, Meiji University, Kanagawa, Japan

**Keywords:** β-tricalcium phosphate, poly(lactic-co-glycolic acid) particles, chelate-setting cement, calcium-phosphate cement, bioresorbability, mechanical property, 30 Bio-inspired and biomedical materials

## Abstract

Calcium-phosphate cements (CPCs) have been used as bone filling materials in orthopaedic surgery. However, CPCs are set using an acid-base reaction, and then change into stable hydroxyapatite (HAp) in a living body. Therefore, we developed bioresorbable chelate-setting *β*-tricalcium phosphate (*β*-TCP) cements based on surface modifications of inositol phosphate (IP6). In order to improve the bioresorbability, we fabricated IP6/*β*-TCP cements hybridized with poly(lactic-co-glycolic acid) (PLGA) particles as a pore-forming agent. The compressive strengths of the cements with the amounts of 5 and 10 mass% PLGA particles were 23.2 and 22.8 MPa, respectively. There was no significant difference from cements without PLGA (23.4 MPa). The setting times of the cement specimens with PLGA particles (30 min) were a little longer than those without PLGA particles (26.3 min). The lack of cytotoxicity of the cement specimens was confirmed using osteoblast-like cells (MC3T3-E1). Cylindrical defects were made by drilling into the tibia of mini-pigs and injecting the prepared cement pastes into the defects. Twelve weeks after implantation the specimens were stained with toluidine blue and histologically evaluated. Histological evaluation of cement specimens with PLGA particles showed enhanced bioresorbability. Newly-formed bone was also observed inside cement specimens with PLGA particles. The IP6/*β*-TCP cement specimens with PLGA particles had excellent material properties, such as injectability, compressive strength, high porosity, no cytotoxicity *in vitro*, bioresorption and bone formation abilities *in vivo*. Organic-inorganic hybridized CPCs are expected to be valuable as novel biodegradable paste-like artificial bone fillers.

## Graphical abstract

**Figure:**
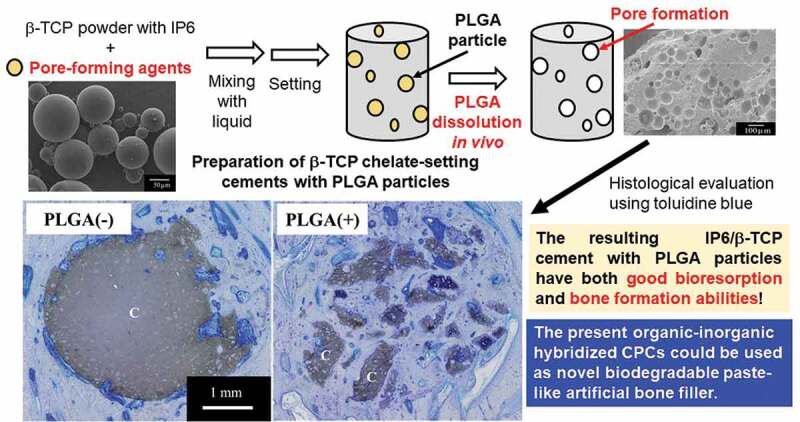

## 1. Introduction

Autologous bone grafts, are generally used to treat bone diseases, and produce good clinical results. However, the amount of bone which can be collected is limited, and may cause further issues for the patient, such as secondary invasion. The development of artificial bone grafts is therefore desirable, to avoid these disadvantages.

Hydroxyapatite (Ca_10_(PO_4_)_6_(OH)_2_; HAp) has been used in bone grafting as a bioactive ceramic, and β-tricalcium phosphate (β-Ca_3_(PO_4_); β-TCP) has been used as a bioresobable ceramic. Injectable calcium-phosphate cement (CPC) pastes have received considerable attention, because they facilitate the use of minimally invasive treatments for patients [1–3]. There are excellent review articles by Ishikawa et al. [4] and Chen et al. [5] on the development of these CPCs. In addition, Medvecky et al. have recently proposed a novel cement that uses phytic acid and phytase as a mixing solution in a two-component CPC raw powder consisting of tetracalcium phosphate (Ca_4_O(PO_4_)_2_) and monetite (CaHPO_4_) [6]. Their CPC is unique in that it uses the enzymatic reaction of phytic acid and phytase.

However, CPCs are set by acid-base reactions, and then changed into stable HAp in a living body. The disadvantages of CPCs are their potential to elicit an inflammatory response, which may be caused by the acid-base reaction, and the low bioresorbability of HAp, which remains in a living body for a long time [7].

To address these issues, we developed bioresorbable, chelate-setting *β*-TCP cements based on the chelate-setting mechanism of inositol phosphate (C_6_H_6_(OPO_3_H_2_)_6_; IP6) [8,9]. The IP6 is also called phytic acid. This newly-developed cement, IP6/β-TCP cement, was fabricated by mixing sodium phosphate solution and ball-milled β-TCP powders surface-modified with IP6. IP6 is present in wheat, rice, corn, and soybean, and strongly chelates calcium ions [10]. In a previous study, we found that chelate-setting IP6/β-TCP cement resorbed about 20% of cement specimens, using a model of tibia defect in eight-week-old pigs [11]. However, in surgery, greater resorbability is needed. The presence of many pores of suitable sizes in the CPCs means that they have high bioresorbability [12,13]. Therefore, we focused on pore-forming agents to increase the resorption of cement specimens.

Our previous study using the pig tibia defect model showed that cements with gelatin particles have high bioresorbability and bone formation eight weeks after implantation [14–16]. These results assumed that the bone-forming ability of cement specimens with gelation particles is higher than that of the CPC which is often used in Japan (Biopex®, HOYA Technosurgical Co. Ltd., Japan). Cements with gelatin also showed a high resorption rate, of about 68.5%, because of the increase in the surface area by the formation of pores. The formation of pores appears to have accelerated the resorption of β-TCP components by the cells. Pore formation from the gelatin particles improves the bioresorbability of the cement specimens, and allows for new bone formation. However, the compressive strength of the CPC with gelatin particles was low, at around 8.6 MPa.

Poly(lactic-co-glycolic acid) (hereafter, PLGA) is a bioresorbable polymer. In this study, we focused on PLGA particles as pore-forming agents, instead of gelatin particles. The addition of PLGA to conventional CPCs gave excellent results with respect to material properties and bioresorbability [17]. Several studies have reported that a pore size of around 100 μm is effective for bone formation [18]. Therefore, we focused on PLGA particles of about 100 μm, for the development of novel cement specimens.

Our final goal was to develop a novel injectable CPC with manipulable mechanical properties and bioresorbability, using the chelate-setting mechanism involving IP6. In this study, we fabricated IP6/β-TCP cements with PLGA particles of 5, 10 and 20 mass%, and evaluated the mechanical properties and bioresorbability of the PLGA-hybridized CPCs. Cytotoxicity tests of the cement specimens were performed using osteoblast-like cells of the MC3T3-E1 cell line. The CPCs were implanted into pig tibial defects for eight weeks in order to optimize the composition of the CPC in terms of its bioresorbability and material properties.

## 2. Materials and methods

### 2.1. Preparation and characterization of PLGA particles

PLGA particles were prepared according to a method previously published by Habraken et al. [19]. The PLGA particles were prepared using a water-in-oil-in-water (w/o/w)-double emulsion solvent evaporation technique. One gram of PLGA (Sigma-Aldrich, weight-average molecular weight (*M*_w_): 34,000–54,000, lactic acid to glycolic acid ratio of 50:50 [w/w]) powder was dissolved in 4 cm^3^ of chloroform inside a 50 cm^3^ tube. To this tube, 0.5 cm^3^ of pure water was added while vortexing vigorously for 90 s. Subsequently, 6 cm^3^ of a 0.3 mass% polyvinyl alcohol (PVA) solution was added, and then vortexed for 90 s. The content of the 50 cm^3^ tube was transferred to a 1000 cm^3^ beaker containing 2 mass% isopropylic alcohol solutions during stirring, and another 394 cm^3^ of 0.3 mass% PVA was added slowly. The suspension was stirred for 1 h. The particles were allowed to settle for 60 min and then the solution was decanted. Ten milliliters of pure water was added, the spheres were washed, centrifuged at 900 rpm for 2 min, and the solution was aspirated. Finally, the PLGA spheres were frozen, and then freeze-dried for 2 h.

PLGA particles were observed using a scanning electron microscope (SEM) (JSM6390LA, JEOL Ltd, Japan) at an accelerating voltage of 10 kV. Samples for SEM observation were coated with platinum using an ion sputtering device.

The sizes of the PLGA particles was measured using a laser scattering particle size distribution analyzer (LA-300, Horiba Ltd., Japan), and the median size was calculated. Prior to measurement, about 0.05 g of prepared powder was dispersed by ultrasonication in 250 cm^3^ of pure water for 3 min. In order to confirm the change in molecular weight of PLGA before and after particularization, the *M*_w_ of the PLGA particles was determined using gel permeation chromatography (GPC) using a TOSOH HLC-8320GPC, UV system (Japan). The GPC measurements were carried out at 40°C using tetrahydrofuran (THF) as an eluent at a flow rate of 1.0 cm^3^·min^−1^. The *M*_w_ was estimated from a calibration curve derived from polystyrene standards. The *M*_w_ of the commercial PLGA product before particularization was 46,000.

### 2.2. Preparation and characterization of starting CPC powders

Starting CPC powders were prepared using the protocol described in our previous report [9]. Ten grams of commercially available β-TCP (β-TCP-100, Taihei Chemical Industrial Co. Led., Japan) powder was placed into a 3000 ppm 40 cm^3^ IP6 (Fujifilm Wako Pure Chemical Industries. Co., Japan) solution in a zirconia pot. The mixture was simultaneously ground and surface-modified using a planetary ball mill (Pulverisette 6, Fritsch Japan Co. Ltd., Japan) under the following conditions: i) a ZrO_2_ pot with a volume of 250 cm^3^, ii) 180 g of ZrO_2_ beads with a diameter of 2 mm, iii) a rotation rate of 300 rpm for 3 h. The resulting slurry was then filtered and freeze-dried for 24 h. The resulting powders were sieved through a 150 μm mesh. This sample was denoted ‘IP6/β-TCP’. As shown in Table 1, the IP6/β-TCP powders obtained were mixed with various weights of PLGA particles at a ratio of IP6/β-TCP:PLGA of 100:0, 95:5, 90:10, and 80:20 [mass%:mass%] for 5 min using a V-shaped mixer (MC, Tsutsui Scientific Instruments Co., Ltd., Japan). The resulting powder mixtures are labelled in this report by the amount of added PLGA. For example, PLGA added to IP6/β-TCP powder is denoted ‘PLGA-cement(x), x: mass%.’

**Table 1.** Composition of cement specimens

Sample	IP6/β-TCP/mass%	PLGA particles/mass%
TCP-cement	100	0
PLGA(5)-cement	95	5
PLGA(10)-cement	90	10
PLGA(20)-cement	80	20

### 2.3. Preparation of mixing solution for cement pastes

To prepare the mixing solution, 2.5 g of disodium hydrogen phosphate (Na_2_HPO_4_), 1.0 g of sodium alginate (C_6_H_7_O_6_Na)_n_) and 1.5 g of citric acid anhydride (C_6_H_8_O_7_) were added in this order to 95 g of pure water. The role of each component is as follows: disodium hydrogen phosphate shortens the setting time, sodium alginate enhances the anti-washout property, and citric acid anhydride improves the dispersibility of β-TCP, to improve the consistency of the final cement [20]. The pH of the solution was adjusted to 7.0 using NaOH solution. All reagents were purchased from Wako Pure Chemical Industries, Ltd., Japan and used as-received, without further purification.

### 2.4. Preparation and characterization of cement pastes

The cement pastes were prepared according to the methods reported in our previous study [9,11]. We mixed the resulting powder with mixing solution in an appropriate powder (P) – liquid (L) ratio for 2 min (P/L ratio = 1.0/0.9 [g/cm^3^]). The initial setting time (IST) was measured using a light Gillmore needle (113.4 g), in accordance with JIS T 0330–4. The prepared cement pastes were packed in plastic molds 8 mm in diameter and 2 mm in height, and the IST was measured at the desired times until the cement pastes were set.

### 2.5. Material properties of cement specimens

test the compressive strength (CS) of the compounds, the cement pastes (P/L ratio = 1.0/0.9 [g/cm^3^]) were packed into cylindrical Teflon molds® 6 mm in diameter and 12 mm in height, maintained at 37°C and at 100% humidity for 24 h. CS tests were performed on the set cement specimens using a universal testing machine (AG-5KNXplus, Shimadzu Co., Kyoto, Japan). The crosshead speed was 0.5 mm·min^–1^, and a load cell of 5 kN was used. Four cement specimens were tested to obtain an average value and standard deviation.

The X-ray diffraction (XRD) patterns of samples were determined using an X-ray diffractometer (Ultima IV, Rigaku Co., Japan) equipped with a Cu Kα radiation source. Data were collected in the range of 2θ = 10 to 50°, with a step size of 0.02°, and a counting time of 1.2 s·step^−1^. The crystalline phases were identified using the International Centre for Diffraction Data-Powder Diffraction File PDF (ICCD-PDF) for β-TCP (#09-0169) and HAp (#09-0432).

In order to create a model in which PLGA particles are completely dissolved *in vivo*, set cement specimens with PLGA were burned at 600°C for 4 h in a tube furnace (KOYO thermos systems CO., LTD). PLGA is well known to burn out completely at a pyrolysis point of 263°C. The microstructure of the cement specimens with PLGA particles were observed using SEM. We determined the true density of the CPCs using a pycnometer, and assessed the bulk density by caliper. We calculated the porosity using Equation 1.

(1) $$Porosity\;\;\left[\%\right]=100-\;\frac{Bulk\;\;\;Density\;\left[\frac{g}{cm^{3}}\right]}{True\;\;\;Density\;\left[\frac{g}{cm^{3}}\right]}\times 100$$

### 2.6. Solubility of cement specimens

In order to investigate the solubility of the cement specimens, around 0.5 cm^3^ of the pastes (P/L ratio = 1.0/0.9 [g/cm^3^]) were immersed in 10 cm^3^ of simulated body fluid (SBF) [21,22] by syringe over a period of 2 to 12 weeks. The SBF was replaced every week. At the desired time points, the lactic acid concentration in the solution released from the cement specimens was determined using a lactic acid measuring instrument (Lactate Pro2, ARKRAY, Japan). The pH measurement was carried out using a 50 cm^3^ tube. No solution exchange was performed in this tube. On the last day, the cement specimens were dried and the microstructure observed using SEM.

### 2.7. Cytotoxicity of cement specimens

To examine the cytotoxicity of the cement specimens, we performed cell culture tests using MC3T3-E1 osteoblastic cells. The cement paste was placed into in a plastic mound with a diameter of 4 mm and a height of 8 mm, and incubated at 37°C for 24 h. After setting, the paste was sterilized using an ethylene oxide gas sterilizer (CT-190 C, Toho Co. Ltd., Japan). Cells were seeded into 12-well plates at a density of 6 × 10^4^ cells/well. After 24 h, the cell culture medium of 1 cm^3^ was replaced, and the insert (Transwell®, Corning, USA) containing the cement specimen was set in the well plate. Medium (0.8 cm^3^) was added to the insert. The cells were then cultured for another one or three days. Following culture, the number of cells was counted. A 12-well polystyrene plate was used as a control.

### 2.8. In vivo evaluation of cement specimens using a pig tibia model

We used male pig weighing 87 kg to evaluate the bioresorbability of the set cement pastes *in vivo*, and assess the subsequent bone formation. The right tibias of the pigs were exposed, and cylindrical defects 4.0 mm in diameter were drilled into the epiphyses of the tibias. The premixed IP6/β-TCP powders, with and without PLGA particles, were sterilized with ethylene oxide gas. The powder was mixed for 2 min with the mixing solutions at the P/L ratio = 1.0/0.9 [g/cm^3^]. The cement pastes (0.15 cm^3^) were packed into plastic syringes, and then injected into the defect sites. The pigs had access to a standard laboratory diet and water throughout the studies. Surgical procedures were performed according to the Guidelines for Animal Care and Use Committee of Meiji University.

Eight weeks after implantation, the pigs were euthanized, and their tibias were harvested. For histological observation, the freshly isolated tibias were fixed with 70% ethanol and then non-decalcified and polished sections were prepared. Histological evaluation was carried by light microscopy out after toluidine blue (TB) staining.

## 3. Results and discussion

### 3.1 Characterization of PLGA particles

**Figure 1.**
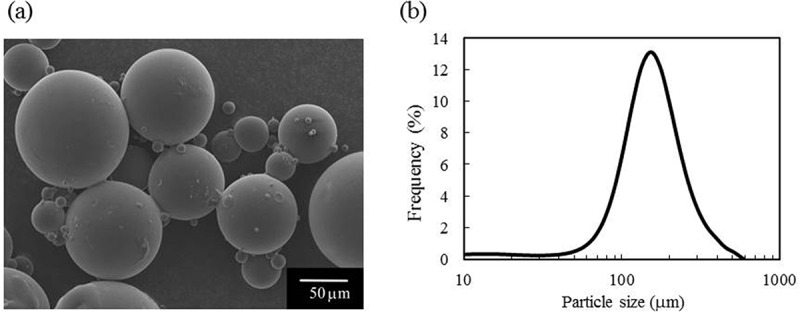
Properties of the PLGA particles: (a) SEM image, and (b) particle size distribution

Figure 1(a) shows the morphology of the PLGA particles, as visualized using SEM. Figure 1(b) shows the particle size distribution of the PLGA particles. The particle sizes ranged from 50 to 200 μm, with a median diameter of 126.4 μm and a mode of 128.9 μm (*n* = 4).

Pores of 50–200 μm may form in and on the cement specimens by dissolution of the PLGA particles *in vivo*. Macropores of 100 μm or more have been reported to promote bone growth [23]. Thus, the PLGA particles used in this study had a size that allowed osteoblasts and osteoclasts to invade. The presence of these cells may promote bioresorption and bone formation in and around the cement specimens.

The *M*_w_ of the present PLGA particles was 51,000. Low molecular weight polymers are usually easily dissolved. Therefore, we used PLGA particles with this particle size and molecular weight for cement fabrication.

### 3.2. Properties of the CPC pastes

**Figure 2.**
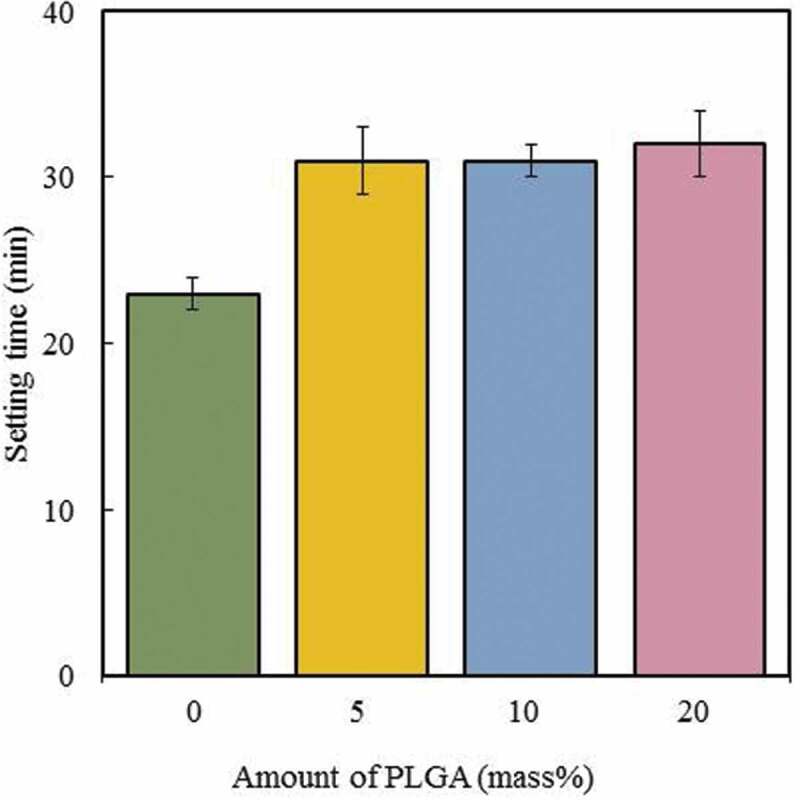
Setting time of the cement paste with PLGA particles. Error bars represent the standard deviation, *n* = 3

Figure 2 shows the IST of cement pastes with PLGA particles were 31 to 32 min. These setting times were longer than those of cements with no additions. This may be because small amounts of PLGA particles were dissolved in the mixing liquid during kneading, which delayed the setting. The setting time of the PLGA cement depended upon the presence or absence of PLGA particles, rather than the amount added. We postulated that a second component, the PLGA particles, may be inhibiting the chelate-setting of IP6.

In clinical applications, it is very important that cement pastes have a moderate IST, of about 10–15 min [2,24]. For the clinical application of our cement in the near future, we need to reduce the setting time. One approach to reduce the setting time is to increase the amount of Na_2_HPO_4_ in the mixture. Nagata et al [20] reported that Na_2_HPO_4_ plays a role in shortening the setting time of cement paste; however, it also decreases the compressive strength. Thus, it is necessary to balance the material properties. In addition to the above, the setting time is proportional to the powder-liquid ratio [25]. Therefore, there is a possibility to improve the method by using a small amount of liquid to increase the viscosity.

### 3.3. Material properties of the CPC cements

**Figure 3.**
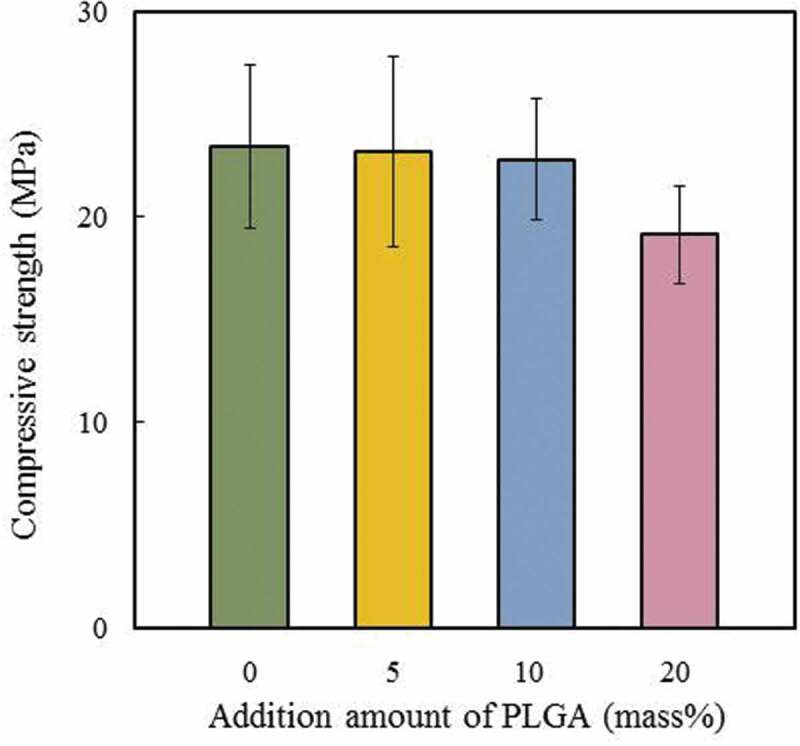
Compressive strength of cement specimens with and without PLGA particles. Error bars represent the standard deviation, *n* = 6

Figure 3 shows the CS of the cement specimens with and without PLGA particles. The CS of the PLGA(0)-cement was 23.42 MPa. The strengths of PLGA(5)- and PLGA(10)-cements were 23.17 MPa and 22.77 MPa, respectively. There was no significant difference in the CS of the cements with and without PLGA particles. However, the strength of PLGA(20)-cement was 19.1 MPa; this value was significantly lower than those of the other specimens. Therefore, we judged that CS was not significantly affected by the addition of a small amount of PLGA particles (5 or 10 mass%). The addition of 20 mass% of PLGA particles reduced the CS of the cement specimens. Zheng et al. reported that the strength of their materials increased with increasing amounts of PLGA [26]. They contended that the strength was improved by the following reaction between lactic acid dissolved from PLGA produced by hydrolysis and Ca^2+^ ions derived from cement specimens.

(2) $$C{a^{2+}}^{\;}+2CH_{3}CHOHCO{O^{-}}^{\;}\to \;Ca{\left(CH_{3}CHOHCOO\right)}_{2}$$

Habraken et al. found that the addition of a second component, such as PLGA particles, may reduce the CS of the material [19]. We considered that the CS of our specimens was comparable to that of the control, due to the simultaneous increase and decrease in strength caused as described above. The CS of the tibia is 15 MPa to withstand compressive fracture. We confirmed that the required value could be achieved by adding 5 or 10 mass% of PLGA particle in this experiment.

**Figure 4.**
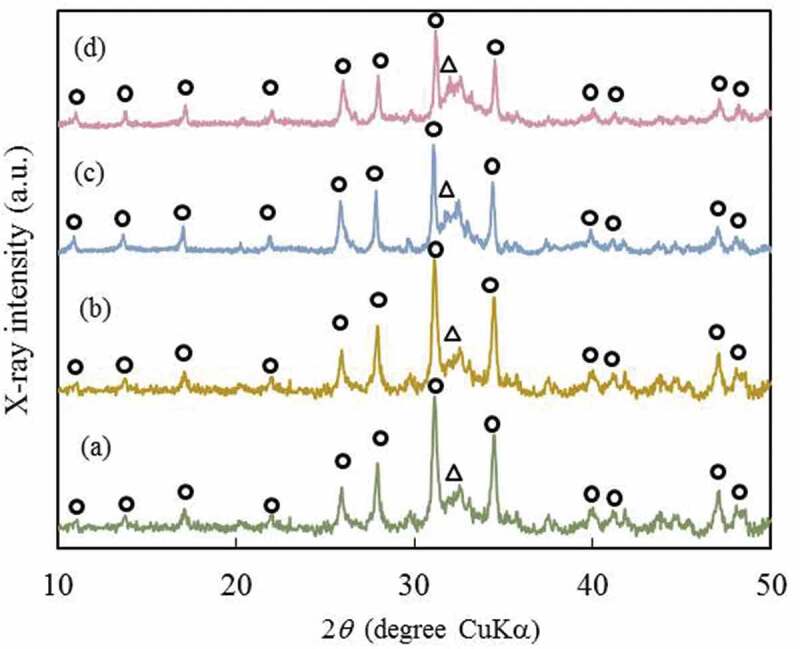
XRD patterns of the cement specimen with different amounts of PLGA particles: (a) 0, (b) 5, (c) 10 and (d) 20 mass%. Open circle and triangle indicate typical β-TCP and HAp peaks, respectively

Figure 4 shows the XRD patterns of cement specimens after CS testing. No significant difference was observed between the cement specimens with and without PLGA particles. In all of the cement specimens, the main crystalline phase was β-TCP. We assumed that these cement specimens are bioresorbable, due to their main crystalline phase being β-TCP which, is highly soluble, compared to the stable HAp phase.

These results suggest that the addition of PLGA particles does not adversely affect the characteristics of chelate-setting CPC, in which the main crystalline phase remains as the original material.

**Figure 5.**
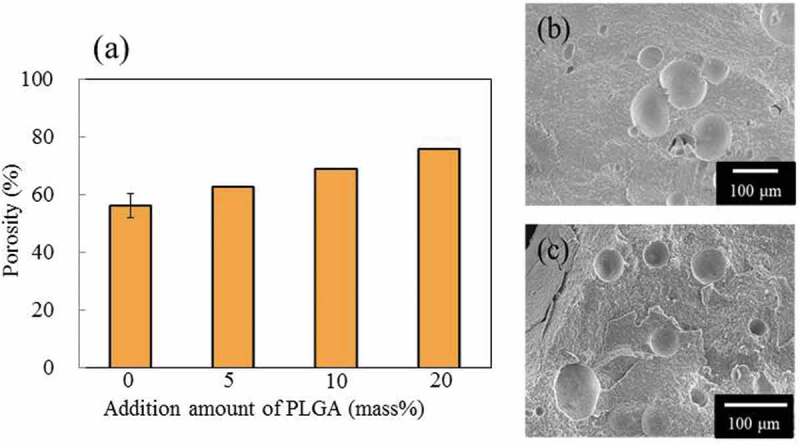
Properties of cement specimens before and after firing at 600°C for 4 h: (a) porosities of cement specimens, *n* = 2, (b, c) SEM images of PLGA(10) before and after firing, respectively

Figure 5(a) shows the porosity of cement specimens on the basis of a PLGA-burned out model. These data showed that the porosity increased in proportion to the amount of PLGA added. The results from the duplicate experiments were in good agreement, showing 2% difference from the first experiment for the specimens with PLGA added. In another study, we reported that bioceramics with a porosity of 70% produced osteoinduction after implantation in porcine muscle [27]. The porosity of the cement specimens in this study were also generally around 70%, and the specimens were therefore expected to show osteoinduction.

Figure 5(b,c) show SEM images before and after burning the specimens with 10 mass% of PLGA particles out at 350°C for 4 h. It is clear from Figure 5(c) that macro pores of size 50 to 200 μm were formed by burning out PLGA particles.

### 3.4. Solubility of cements specimens

**Figure 6.**
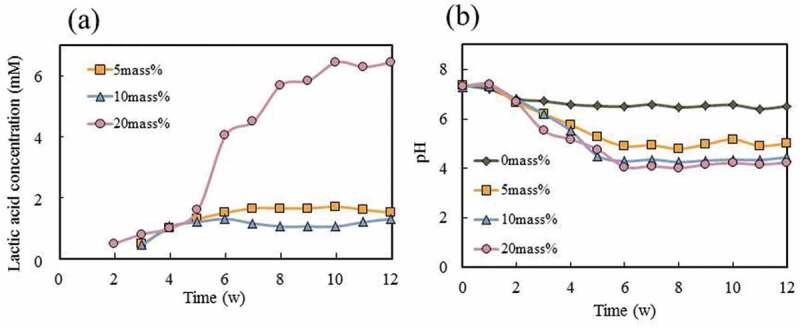
Solubility test of cement specimens with various amount of PLGA particles: (a) lactic acid concentration from cement specimens, (b) changes in pH values during solubility testing (*n* = 2)

Figure 6 shows the results of the solubility tests of the cement specimens with various amounts of PLGA particles in SBF solution over a period of 12 weeks. The error between two trials is small, and the error is within the plot. The lactic acid concentration of cement specimens with 20 mass% PLGA was markedly increased after week 5 (Figure 6(a)). There was no change in lactic concentration at 5 and 10 mass% of PLGA after five weeks. We concluded that no further elution occurs from cement specimens with 5 or 10 mass% PLGA particles. Since PLGA hydrolyses into lactic acid and glycolic acid, the lactic acid concentration can be used as an indicator of the amount of PLGA degraded from the cement. *In vivo* there is no difference in the dissolution rate at five weeks, but the subsequent dissolution rate is expected to be different.

The change in pH value over time caused by the degradation of cement specimens is shown in Figure 6(b). Overall the values obtained from a duplicate experiment showed less than 2% variation from the initially obtained values. The addition of PLGA particles caused the SBF to become acidic after two weeks. This effect may be due to the lactic acid and glycolic acid derived from PLGA. The pH values of the cement specimens with PLGA particles had decreased rapidly by the sixth week and then remained stable. The final pH value of 4.06 was reached after six weeks of immersion, following which there was very little change (PLGA(20)-cement).

The decrease of the initial pH may occur because of the hydrolysis of PLGA. Compared to cement specimens with 10 mass% and 20 mass% PLGA particles, the pH value of PLGA(5)-cement was slightly higher than that of PLGA(10) or (20) cements. The pH value of solution was therefore dependent upon concentration of lactic acid from the PLGA particles. We considered that the lactic acid in the SBF was saturated in the case of cement specimens with PLGA particles of 10 mass% or more. These results indicate that the PLGA in the CPCs dissolves in two weeks or longer. This degradation rate is considered to be modest, as osteoclasts resorb bone over two to four weeks. The degradation of PLGA particles may lead to the formation of macropores inside the cement specimens. Therefore, the amount of lactic acid detected may increase, depending upon the number of PLGA particles.

**Figure 7.**
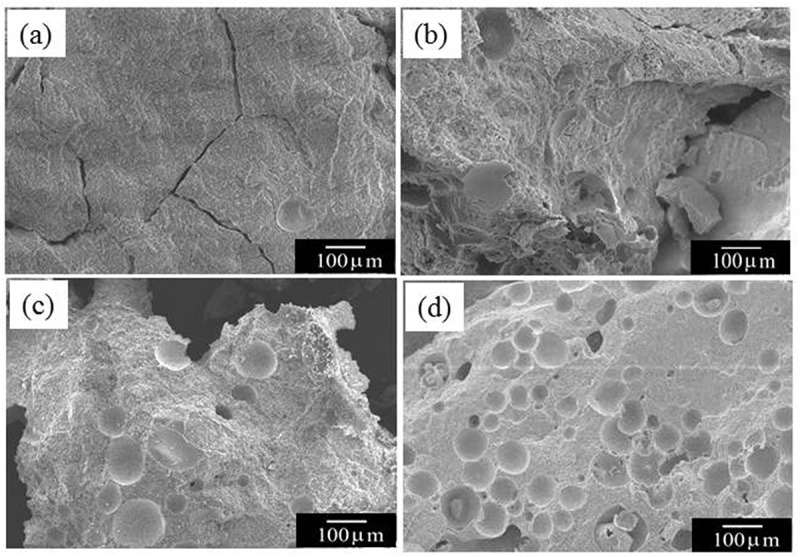
SEM images of cement specimens with PLGA particles of 0 mass% (a), 5 (b), 10 (c) and 20 (d) after 12 weeks of immersion in SBF

Figure 7 shows SEM images of cement specimens with various amounts of PLGA. Pores could be confirmed in all samples to which PLGA particles had been added. The number of pores is proportional to the amount of PLGA particles added. Especially, the PLGA(20)-cement had many pores (Figure 7(d)).

### 3.5. In vitro evaluation of cement specimens

**Figure 8.**
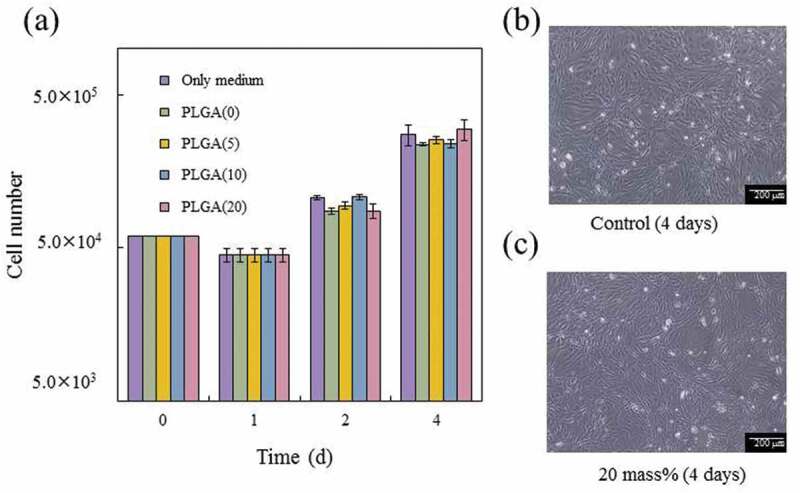
Cytotoxicity of cement specimens in MC3T3-E1 cells: (a) cell proliferation; error bars represent the standard deviation, *n* = 3, (b) morphologies of cells cultured on the control, and (c) morphologies of cells co-cultured with the PLGA(20)-specimens

The results of the cytotoxicity tests using MC3T3-E1 cells are shown in Figure 8(a). Cells co-cultured with cement specimens showed similar proliferation to the control. Therefore, none of the cement specimens, with or without PLGA particles, showed toxicity. Figure 8(b,c) show typical optical microscopic images of the control and PLGA(20)-cement specimen after four days of cell culture. No abnormalities in cell morphology were observed with the addition of PLGA, and the cells proliferated well compared to the control. Therefore, we clarified that the present β-TCP/PLGA cement did not show cytotoxicity *in vitro* evaluation using mouse-derived MC3T3-E1 cells.

### 3.6. In vivo evaluation of cement specimens

**Figure 9.**
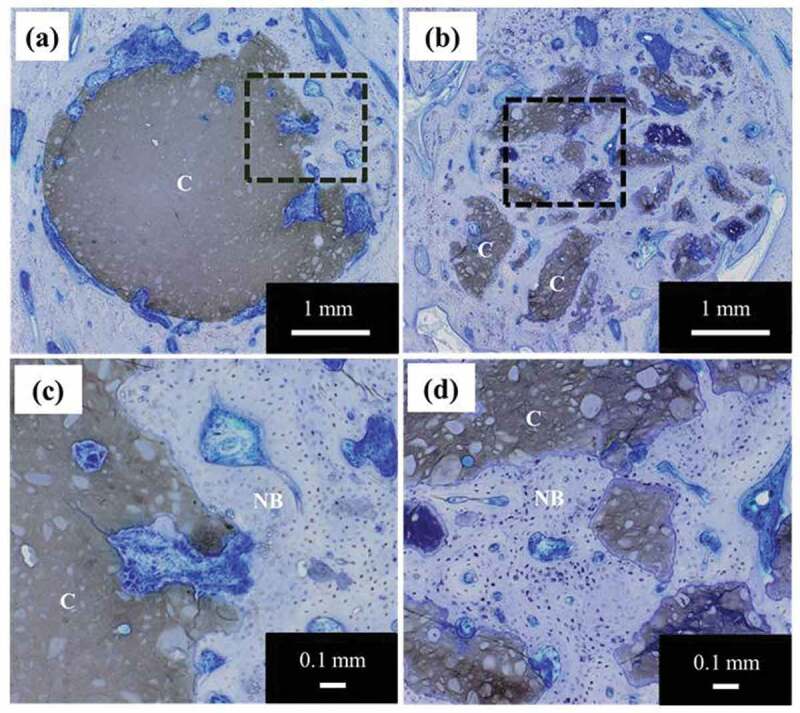
Histological evaluation of PLGA-added cements with toluidine blue staining (C: remaining cement specimens, NB: new bone): (a) cement specimens without PLGA, (b) cement specimens with 10 mass% PLGA particles, (c) high magnification image of the area circled by the square in (a), and (d) high magnification image of the area circled by the square in (b)

Histological images of the TB staining of a pig’s tibia with cement specimens are shown in Figure 9. Figure 9(a,b) show histological images of cement specimens with and without PLGA particles, respectively. Magnified views of the area within the dotted line in Figure 9(a,b) are shown in Figure 9(c,d), respectively. We observed degradation in all of the cement specimens. This degradation may be due to the formation of pores derived from PLGA particles. We also observed new bone formation in both samples. New bone formation occurred inside the PLGA(10)-cement; however, in the case of β-TCP-cement, bone formation occurred only at the interface between the host bone and the specimen. These results demonstrate that cement specimens with 10 mass% PLGA particles had both good bioresorption and high bone formation abilities.

As shown in Figure 7, a pore size of about 100 μm was observed using SEM. We concluded that osteoclasts passed through the pores and promoted the degradation of the material. The improvement in material resorption may be attributed to the enhanced solubility of cement due to the decrease of pH in the presence of osteoclasts.

## 4. Conclusions

The IST of the cement pastes with PLGA particles was 31–32 min. These setting times have to be shorter, to increase handling ability. The CS of the cement with PLGA produced strength of 15 MPa or more, corresponding to the CS of a compression fracture of the cervical spine, for all amounts of PLGA. The added PLGA particles were degraded two weeks after immersion in SBF, a model of the body chemistry. From the SEM images and the porosity results, we confirmed that the product has both high solubility and clinically acceptable CS. PLGA-cement specimens had no cytotoxicity. *In vivo* studies revealed that parts of both cements were in direct contact with the host and newly-formed bones. Resorption of the surface and interior of the cement specimen with PLGA particles was confirmed.

The IP6/*β*-TCP cement specimens with PLGA particles have excellent material properties, including good bioresorption and bone formation abilities. Therefore, the present organic-inorganic hybridized CPCs could be used as novel biodegradable paste-like artificial bone filler.
